# Potential Threats to Human Health from Eurasian Avian-Like Swine Influenza A(H1N1) Virus and Its Reassortants

**DOI:** 10.3201/eid2807.211822

**Published:** 2022-07

**Authors:** Shuai-Yong Wang, Feng Wen, Ling-Xue Yu, Juan Wang, Man-Zhu Wang, Jie-Cong Yan, Yan-Jun Zhou, Wu Tong, Tong-Ling Shan, Guo-Xin Li, Hao Zheng, Chang-Long Liu, Ning Kong, Guang-Zhi Tong, Hai Yu

**Affiliations:** Chinese Academy of Agricultural Sciences, Shanghai, China (S.-Y. Wang, L.-X. Yu, J. Wang, M.-Z. Wang, J.-C. Yan, Y.-J. Zhou, W. Tong, T.-L. Shan, G.-X. Li, H. Zheng, C.-L. Liu, N. Kong, G.-Z. Tong, H. Yu);; Foshan University, Foshan, Guangdong, China (F. Wen).

**Keywords:** influenza, avian-like swine influenza A(H1N1), swine flu, respiratory infections, viruses, zoonoses, China, H1N1

## Abstract

During 2018–2020, we isolated 32 Eurasian avian-like swine influenza A(H1N1) viruses and their reassortant viruses from pigs in China. Genomic testing identified a novel reassortant H3N1 virus, which emerged in late 2020. Derived from G4 Eurasian H1N1 and H3N2 swine influenza viruses. This virus poses a risk for zoonotic infection.

Since emerging in 2001, Eurasian avian-like (EA) swine influenza A(H1N1) virus has gradually become the predominant lineage and continues to circulate among pigs in China (*1*–*3*). Introduction of the 2009 pandemic H1N1 virus (pH1N1) among pigs has increased its reassortment with EA H1N1 swine influenza A viruses (IAVs), and several reassortant variants with the potential to infect humans have been detected in China (*4*–*8*). 

Multiple genotypes have been identified in EA H1N1 swine IAVs from pigs in China, and recent data suggest that the potentially pandemic genotype 4 (G4) reassortant has predominated among swine populations in China since 2016 (*4*,*8*). To clarify their prevalence and genotype characterizations, we isolated 32 swine IAVs in China during 2018–2020, including 6 novel reassortant H3N1 viruses that carry the hemagglutinin (HA) gene derived from human H3N2 lineage, and conducted phylogenic analysis of 8 gene segments from these viruses. 

## The Study

During January 2018–December 2020, we collected 1,006 swab samples from pigs with symptoms typical of swine influenza, such as fever and cough, on pig farms across 6 provinces (Shanghai, Jiangsu, Zhejiang, Tianjin, Hebei, and Shandong) in China. We isolated viruses using MDCK cells and determined viral whole-genome sequences by Sanger sequencing. We isolated a total of 32 swine IAVs from the 1,006 swab samples, an isolation rate of 3.18% (Table). To determine the phylogenetic evolution of the 32 isolates, we performed genetic analyses using available sequences of related viruses from the GenBank and GISAID (https://www.gisaid.org) databases. 

**Table:** Detailed information of 32 viruses isolated in study of Eurasian avian-like swine influenza A(H1N1) virus and its reassortant viruses, China*

Strain name	Date collected	Place collected	Gene segment								G
			PB2	PB1	PA	HA	NP	NA	M	NS	
A/swine/Shanghai/37/2018	1/2018	Shanghai	EA	EA	EA	EA	EA	EA	EA	EA	1
A/swine/Shanghai/56/2018	1/2018	Shanghai	EA	EA	EA	EA	EA	EA	EA	EA	1
A/swine/Shanghai/72/2018	1/2018	Shanghai	EA	EA	EA	EA	EA	EA	EA	EA	1
A/swine/Shanghai/136/2018	3/2018	Shanghai	EA	EA	EA	EA	EA	EA	EA	EA	1
A/swine/Hebei/11/2018	3/2018	Hebei	EA	EA	EA	EA	EA	EA	EA	EA	1
A/swine/Hebei/47/2018	3/2018	Hebei	EA	EA	EA	EA	EA	EA	EA	EA	1
A/swine/Shandong/8/2018	3/2018	Shandong	pH1N1	pH1N1	pH1N1	EA	pH1N1	EA	pH1N1	pH1N1	2
A/swine/Zhejiang/5/2018	12/2018	Zhejiang	pH1N1	pH1N1	pH1N1	EA	pH1N1	EA	pH1N1	TRIG	4
A/swine/Shandong/20/2018	12/2018	Shandong	pH1N1	pH1N1	pH1N1	EA	pH1N1	EA	EA	TRIG	5
A/swine/Jiangsu/16/2018	12/2018	Jiangsu	pH1N1	pH1N1	pH1N1	EA	pH1N1	EA	EA	TRIG	5
A/swine/Jiangsu/28/2018	12/2018	Jiangsu	pH1N1	pH1N1	pH1N1	EA	pH1N1	EA	EA	TRIG	5
A/swine/Hebei/16/2019	1/2019	Tianjin	pH1N1	pH1N1	pH1N1	EA	pH1N1	EA	pH1N1	pH1N1	2
A/swine/Zhejiang/19/2019	1/2019	Zhejiang	pH1N1	pH1N1	pH1N1	EA	pH1N1	EA	pH1N1	TRIG	4
A/swine/Shandong/66/2019	1/2019	Shandong	pH1N1	pH1N1	pH1N1	EA	pH1N1	EA	pH1N1	TRIG	4
A/swine/Shandong/73/2019	1/2019	Shandong	pH1N1	pH1N1	pH1N1	EA	pH1N1	EA	pH1N1	TRIG	4
A/swine/Jiangsu/91/2019	11/2019	Jiangsu	pH1N1	pH1N1	pH1N1	EA	pH1N1	EA	pH1N1	TRIG	4
A/swine/Zhejiang/22/2019	12/2019	Zhejiang	pH1N1	pH1N1	pH1N1	EA	pH1N1	EA	EA	TRIG	5
A/swine/Heibei/66/2019	12/2019	Hebei	pH1N1	pH1N1	pH1N1	EA	pH1N1	EA	EA	TRIG	5
A/swine/Tianjin/27/2019	12/2019	Tianjin	pH1N1	pH1N1	pH1N1	EA	pH1N1	EA	EA	TRIG	5
A/swine/Tianjin/50/2020	10/2020	Tianjin	pH1N1	pH1N1	pH1N1	EA	pH1N1	EA	pH1N1	TRIG	4
A/swine/Tianjin/82/2020	10/2020	Tianjin	pH1N1	pH1N1	pH1N1	EA	pH1N1	EA	pH1N1	TRIG	4
A/swine/Zhejiang/25/2020	10/2020	Zhejiang	pH1N1	pH1N1	pH1N1	EA	pH1N1	EA	pH1N1	TRIG	4
A/swine/Jiangsu/93/2020	10/2020	Jiangsu	pH1N1	pH1N1	pH1N1	EA	pH1N1	EA	pH1N1	TRIG	4
A/swine/Shandong/88/2020	10/2020	Shandong	pH1N1	pH1N1	pH1N1	EA	pH1N1	EA	pH1N1	TRIG	4
A/swine/Tianjin/121/2020	11/2020	Tianjin	pH1N1	pH1N1	pH1N1	EA	pH1N1	EA	EA	TRIG	5
A/swine/Jiangsu/100/2020	11/2020	Jiangsu	pH1N1	pH1N1	pH1N1	EA	pH1N1	EA	EA	TRIG	5
A/swine/Zhejiang/76/2020	12/2020	Zhejiang	pH1N1	pH1N1	pH1N1	H3	pH1N1	EA	pH1N1	TRIG	nH3N1
A/swine/Zhejiang/83/2020	12/2020	Zhejiang	pH1N1	pH1N1	pH1N1	H3	pH1N1	EA	pH1N1	TRIG	nH3N1
A/swine/Zhejiang/109/2020	12/2020	Zhejiang	pH1N1	pH1N1	pH1N1	H3	pH1N1	EA	pH1N1	TRIG	nH3N1
A/swine/Zhejiang/211/2020	12/2020	Zhejiang	pH1N1	pH1N1	pH1N1	H3	pH1N1	EA	pH1N1	TRIG	nH3N1
A/swine/Zhejiang/269/2020	12/2020	Zhejiang	pH1N1	pH1N1	pH1N1	H3	pH1N1	EA	pH1N1	TRIG	nH3N1
A/swine/Zhejiang/360/2020	12/2020	Zhejiang	pH1N1	pH1N1	pH1N1	H3	pH1N1	EA	pH1N1	TRIG	nH3N1

*EA, Eurasian; G, genotype; HA, hemagglutinin; M, matrix; NA, neuraminidase; NP, nucleoprotein; NS, nonstructural; pH1N1, 2009 pandemic influenza (H1N1); PA, polymerase acidic protein; PB1, polymerase basic protein 1; PB2, polymerase basic protein 2; TRIG, triple reassortant internal gene.

**Figure 1:**
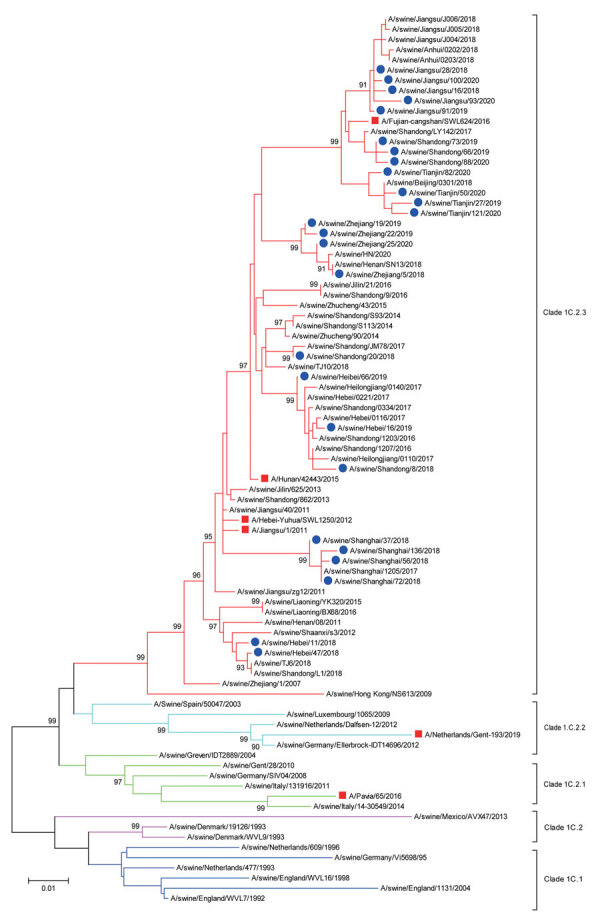
Maximum-likelihood phylogenetic tree of hemagglutinin genes of Eurasian avian-like swine influenza A(H1N1) viruses from pigs on pig farms in 6 provinces of China (blue circles) and reference sequences from humans (red squares). The phylogeny of available sequences of related viruses from GenBank and GISAID database (https://www.gisaid.org) and the 26 HA genes sequenced in this study were inferred by using MEGA version 7 (https://www.megasoftware.net) under the general time-reversible plus gamma distribution model with 1,000 bootstrap replicates. Scale bar indicates substitutions per nucleotide.

**Figure 2:**
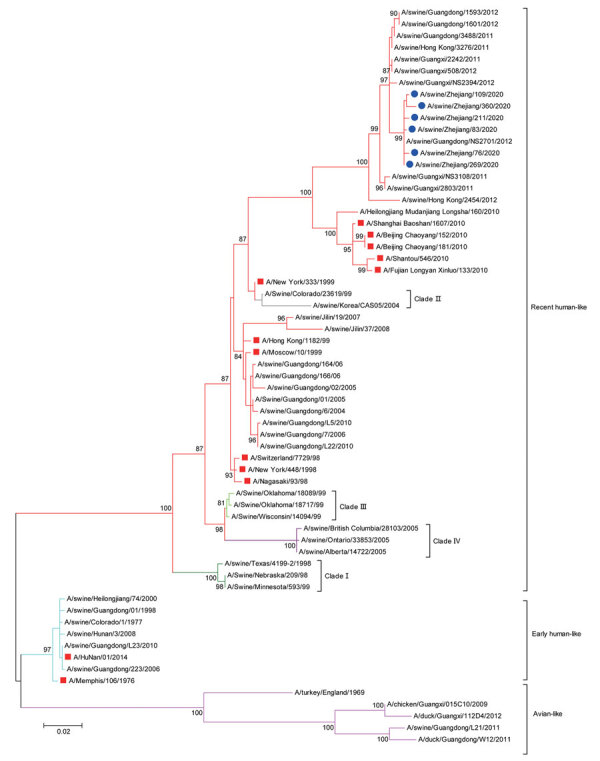
Maximum-likelihood phylogenetic tree of hemagglutinin genes of novel swine influenza A(H3N1) viruses from pigs on pig farms in 6 provinces of China (blue circles) and reference sequences from humans (red squares). The phylogeny of available sequences of related viruses from GenBank and GISAID database (https://www.gisaid.org) and the 6 HA genes sequenced in this study were inferred by using MEGA version 7 (https://www.megasoftware.net) under the general time-reversible plus gamma distribution model with 1,000 bootstrap replicates. Scale bar indicates substitutions per nucleotide.

Phylogenetic analysis revealed that the HA genes of 26 viruses isolated in the study were grouped within clade 1C.2.3 of EA H1N1 lineage (Figure 1). However, the HA genes of the 6 novel reassortant H3N1 viruses were located in the recently circulating human-like H3N2 lineage and shared the highest genetic identity (99.7%–99.9%) with a swine H3N2 virus (A/Swine/Guangdong/NS2701/2012) in China (Figure 2). The neuraminidase (NA) genes of all 32 isolates were grouped within the EA H1N1 lineage (Appendix Figure). On the basis of sequence analysis of the HA and NA genes, we identified the 32 swine IAVs isolated in this study as EA H1N1 (n = 26; isolation rate: 2.58%) and H3N1 (n = 6; isolation rate: 0.6%), indicating that EA H1N1 was the predominant virus subtype circulating among the sampled pig population in China. 

Origins of the 6 internal gene segments, polymerase basic (PB) 1 and 2, polymerase acidic (PA), nucleoprotein (NP), matrix (M), and nonstructural (NS) genes, were remarkably diverse: EA H1N1, pH1N1, and TRIG (triple-reassortant internal gene) lineages (Appendix Figure). Among 6 EA H1N1 swine viruses, all internal gene segments were of EA H1N1 lineage. Among 26 reassortant EA H1N1 and H3N1 viruses, the PB2, PB1, PA, and NP genes all originated from the pH1N1 lineage; the M genes were mainly from both the pH1N1 and EA H1N1 lineages. Almost all NS genes originated from the TRIG lineage, but 2 originated from the pH1N1 lineage. On the basis of phylogenetic analyses of the 8 gene segments, including from HA and NA genes, we identified the viruses isolated in our study as G1 (n = 6), G2 (n = 2), G4 (n = 10), G5 (n = 8), and novel H3N1 (n = 6) viruses, according to the genotype classification existing at that time (*7*,*8*). We isolated viruses year-round to capture seasonal strains. Over the 36-month survey period, G1 viruses disappeared after 2018, G2 viruses were sporadically detected in 2018–2019, and G4 viruses, with an isolation rate of 1.0%, became a predominant genotype beginning in 2018. In our annual surveillance, we found another predominant virus genotype, G5, that had an isolation rate of 0.8%. These results suggest that the internal genes of the pH1N1 lineage had become predominant in contemporary swine IAVs among the pigs in the survey region. 

Of note, in late 2020, we detected the H3N1 swine IAVs in 6 isolates (all from Zhejiang Province), indicating that this is a novel emerging recombinant genotype. Phylogenetic analyses demonstrated that the 6 novel H3N1 reassortant swine IAVs contained NA genes from the EA H1N1; PB2, PB1, PA, NP, and M genes from pH1N1; and NS genes from TRIG swine lineages. This combination is similar to the potentially pandemic G4 viruses except for the HA genes, suggesting that the emergence of novel H3N1 reassortant swine IAVs was a natural reassortant event that derived from G4 and H3N2 swine IAVs.

## Conclusions

Because of their susceptibility to avian, swine, and human IAVs, pigs are regarded as a mixing vessel for generating novel reassortant influenza viruses capable of replicating and spreading among humans (*9*,*10*). Implications for human health reinforce the importance of continuous surveillance of swine IAVs in the pig population. China has the most varied swine influenza virus ecosystem in the world and different subtypes simultaneously circulate among pigs (*11*,*12*). The emergence of potentially pandemic G4 EA H1N1 virus has increased the chances of reassortment with enzootic swine IAVs and the subsequential emergence of novel reassortant swine IAVs. 

We isolated 6 EA H1N1 swine viruses and 26 reassortant EA H1N1 and H3N1 swine viruses in this study. Analysis results indicated that the reassortment of gene segments between EA H1N1 swine viruses and other enzootic swine viruses occurred frequently, and the reassortant swine viruses became established among the sampled pig population. Previous studies have reported several cases of human disease from EA H1N1 swine IAV or its reassortant viruses in Europe and China (*13*–*15*). 

Our study, based on swine epidemiologic data from China, demonstrates that EA H1N1 swine influenza virus and its reassortant viruses circulate in swine populations and pose potential threats to human health. Furthermore, we isolated and documented the genetic evolution of novel reassortant H3N1 viruses between potentially pandemic G4 EA H1N1 and H3N2 swine IAVs. These findings highlight the need for surveillance for novel H3N1 viruses in swine and human populations to enable early interventions to avert outbreaks and protect animal and human health. 

Appendix Additional information from study of Eurasian avian-like swine influenza A(H1N1) virus and its reassortant viruses
