# Supplementary material for: Potential Threats to Human Health from Eurasian Avian-Like Swine Influenza A(H1N1) Virus and Its Reassortants
Source: Emerg Infect Dis. 2022 Jul;28(7):1489–93. doi: 10.3201/eid2807.211822 (PMC9239861; doi:10.3201/eid2807.211822)
Supplement: Appendix — Additional information from study of Eurasian avian-like swine influenza A(H1N1) virus and its reassortant viruses [file 21-1822-Techapp-s1.pdf]

Article DOI: https://doi.org/10.3201/eid2807.211822

# Potential Threats to Human Health from Eurasian Avian-Like Swine Influenza A(H1N1) Virus and Its Reassortants

## Appendix

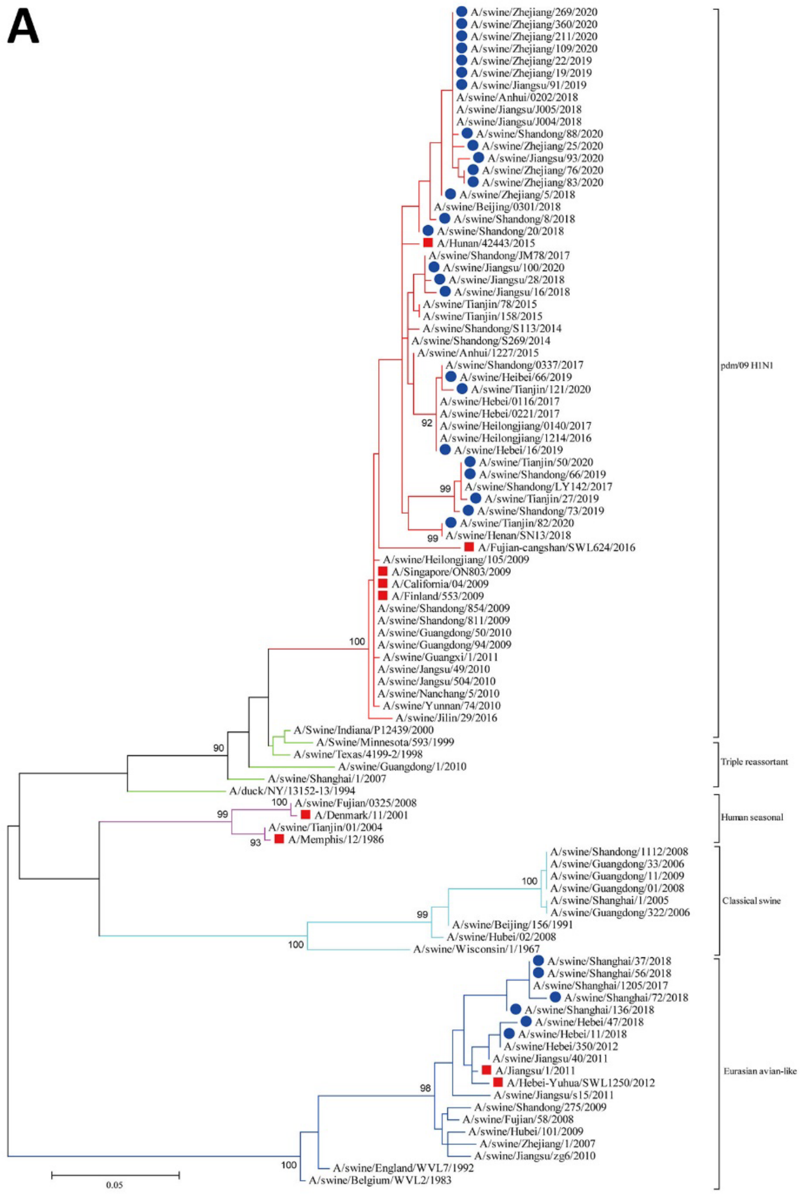

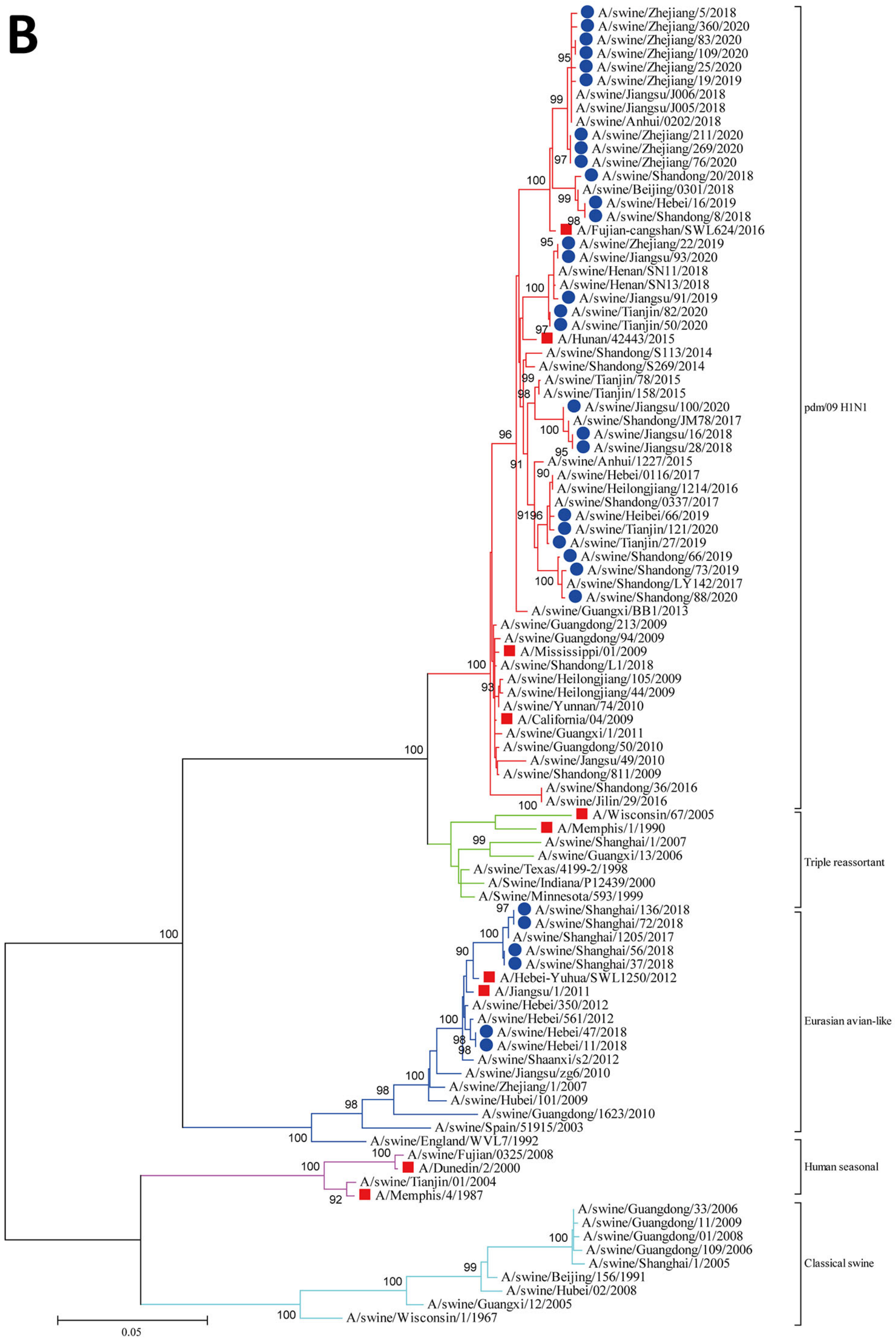
B
A/swine/Zhejiang/5/2018
A/swine/Zhejiang/360/2020
A/swine/Zhejiang/83/2020
A/swine/Zhejiang/109/2020
A/swine/Zhejiang/25/2020
A/swine/Zhejiang/19/2019
A/swine/Jiangsu/J006/2018
A/swine/Jiangsu/J005/2018
A/swine/Anhui/0202/2018
A/swine/Zhejiang/211/2020
A/swine/Zhejiang/269/2020
A/swine/Zhejiang/76/2020
A/swine/Shandong/20/2018
A/swine/Beijing/0301/2018
A/swine/Hebei/16/2019
A/swine/Shandong/8/2018
A/Fujian-cangshan/SWL624/2016
A/swine/Zhejiang/22/2019
A/swine/Jiangsu/93/2020
A/swine/Henan/SN11/2018
A/swine/Henan/SN13/2018
A/swine/Jiangsu/91/2019
A/swine/Tianjin/82/2020
A/swine/Tianjin/50/2020
A/Hunan/42443/2015
A/swine/Shandong/S113/2014
A/swine/Shandong/S269/2014
A/swine/Tianjin/78/2015
A/swine/Tianjin/158/2015
A/swine/Jiangsu/100/2020
A/swine/Shandong/JM78/2017
A/swine/Jiangsu/16/2018
A/swine/Jiangsu/28/2018
A/swine/Anhui/1227/2015
A/swine/Hebei/0116/2017
A/swine/Heilongjiang/1214/2016
A/swine/Shandong/0337/2017
A/swine/Heibei/66/2019
A/swine/Tianjin/121/2020
A/swine/Tianjin/27/2019
A/swine/Shandong/66/2019
A/swine/Shandong/73/2019
A/swine/Shandong/LY142/2017
A/swine/Shandong/88/2020
A/swine/Guangxi/BB1/2013
A/swine/Guangdong/213/2009
A/swine/Guangdong/94/2009
A/Mississippi/01/2009
A/swine/Shandong/L1/2018
A/swine/Heilongjiang/105/2009
A/swine/Heilongjiang/44/2009
A/swine/Yunnan/74/2010
A/California/04/2009
A/swine/Guangxi/1/2011
A/swine/Guangdong/50/2010
A/swine/Jangsu/49/2010
A/swine/Shandong/811/2009
A/swine/Shandong/36/2016
A/swine/Jilin/29/2016
A/Wisconsin/67/2005
A/Memphis/1/1990
A/swine/Shanghai/1/2007
A/swine/Guangxi/13/2006
A/swine/Texas/4199-2/1998
A/Swine/Indiana/P12439/2000
A/Swine/Minnesota/593/1999
A/swine/Shanghai/136/2018
A/swine/Shanghai/72/2018
A/swine/Shanghai/1205/2017
A/swine/Shanghai/56/2018
A/swine/Shanghai/37/2018
A/Hebei-Yuhua/SWL1250/2012
A/Jiangsu/1/2011
A/swine/Hebei/350/2012
A/swine/Hebei/561/2012
A/swine/Hebei/47/2018
A/swine/Hebei/11/2018
A/swine/Shaanxi/s2/2012
A/swine/Jiangsu/zg6/2010
A/swine/Zhejiang/1/2007
A/swine/Hubei/101/2009
A/swine/Guangdong/1623/2010
A/swine/Spain/51915/2003
A/swine/England/WVL7/1992
A/swine/Fujian/0325/2008
A/Dunedin/2/2000
A/swine/Tianjin/01/2004
A/Memphis/4/1987
A/swine/Guangdong/33/2006
A/swine/Guangdong/11/2009
A/swine/Guangdong/01/2008
A/swine/Guangdong/109/2006
A/swine/Shanghai/1/2005
A/swine/Beijing/156/1991
A/swine/Hubei/02/2008
A/swine/Guangxi/12/2005
A/swine/Wisconsin/1/1967
pdm/09 H1N1
Triple reassortant
Eurasian avian-like
Human seasonal
Classical swine
0.05

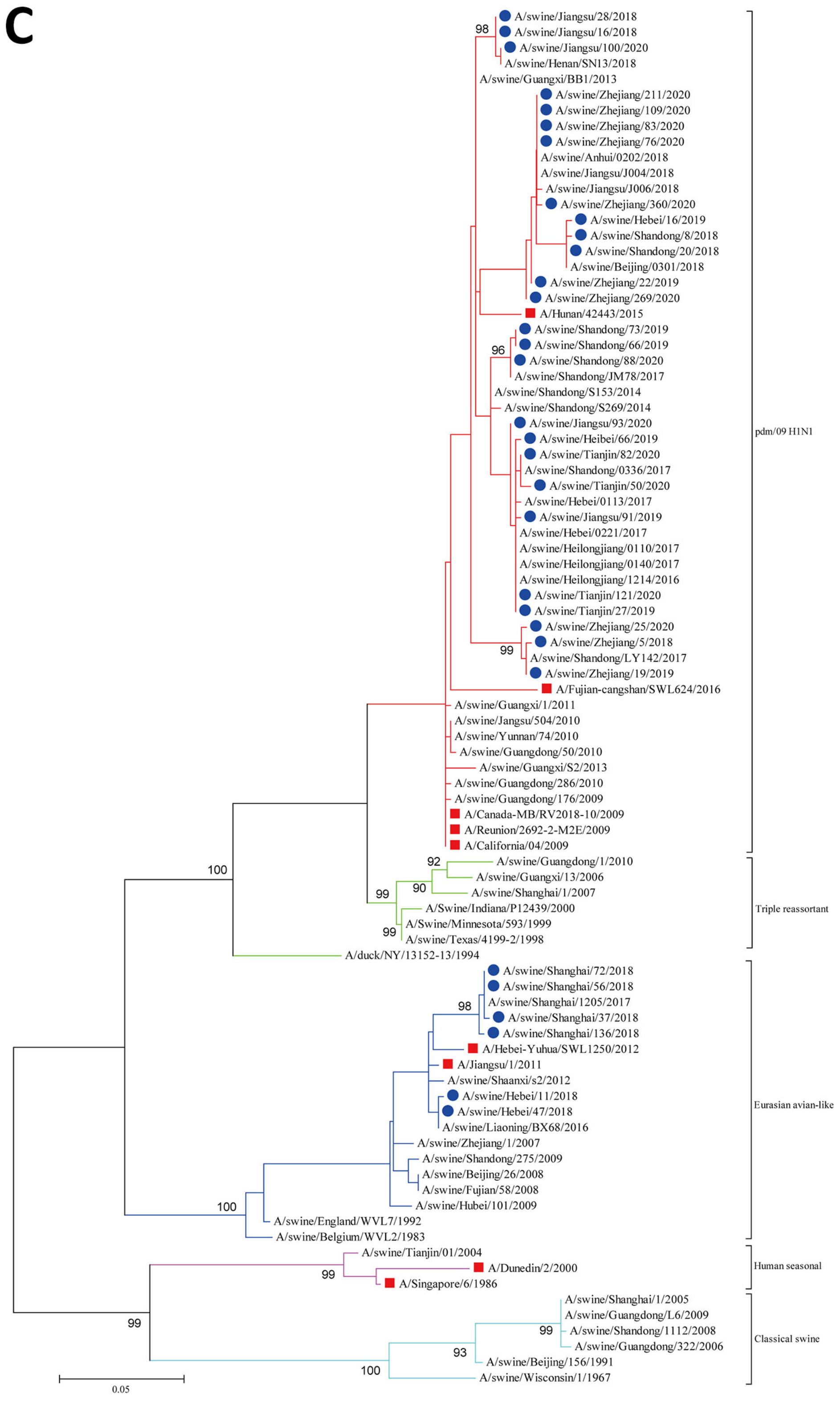
C
A/swine/Jiangsu/28/2018
A/swine/Jiangsu/16/2018
A/swine/Jiangsu/100/2020
A/swine/Henan/SN13/2018
A/swine/Guangxi/BB1/2013
A/swine/Zhejiang/211/2020
A/swine/Zhejiang/109/2020
A/swine/Zhejiang/83/2020
A/swine/Zhejiang/76/2020
A/swine/Anhui/0202/2018
A/swine/Jiangsu/J004/2018
A/swine/Jiangsu/J006/2018
A/swine/Zhejiang/360/2020
A/swine/Hebei/16/2019
A/swine/Shandong/8/2018
A/swine/Shandong/20/2018
A/swine/Beijing/0301/2018
A/swine/Zhejiang/22/2019
A/swine/Zhejiang/269/2020
A/Hunan/42443/2015
A/swine/Shandong/73/2019
A/swine/Shandong/66/2019
A/swine/Shandong/88/2020
A/swine/Shandong/JM78/2017
A/swine/Shandong/S153/2014
A/swine/Shandong/S269/2014
A/swine/Jiangsu/93/2020
A/swine/Heibei/66/2019
A/swine/Tianjin/82/2020
A/swine/Shandong/0336/2017
A/swine/Tianjin/50/2020
A/swine/Hebei/0113/2017
A/swine/Jiangsu/91/2019
A/swine/Hebei/0221/2017
A/swine/Heilongjiang/0110/2017
A/swine/Heilongjiang/0140/2017
A/swine/Heilongjiang/1214/2016
A/swine/Tianjin/121/2020
A/swine/Tianjin/27/2019
A/swine/Zhejiang/25/2020
A/swine/Zhejiang/5/2018
A/swine/Shandong/LY142/2017
A/swine/Zhejiang/19/2019
A/Fujian-cangshan/SWL624/2016
A/swine/Guangxi/1/2011
A/swine/Jangsu/504/2010
A/swine/Yunnan/74/2010
A/swine/Guangdong/50/2010
A/swine/Guangxi/S2/2013
A/swine/Guangdong/286/2010
A/swine/Guangdong/176/2009
A/Canada-MB/RV2018-10/2009
A/Reunion/2692-2-M2E/2009
A/California/04/2009
A/swine/Guangdong/1/2010
A/swine/Guangxi/13/2006
A/swine/Shanghai/1/2007
A/Swine/Indiana/P12439/2000
A/Swine/Minnesota/593/1999
A/swine/Texas/4199-2/1998
A/duck/NY/13152-13/1994
A/swine/Shanghai/72/2018
A/swine/Shanghai/56/2018
A/swine/Shanghai/1205/2017
A/swine/Shanghai/37/2018
A/swine/Shanghai/136/2018
A/Hebei-Yuhua/SWL1250/2012
A/Jiangsu/1/2011
A/swine/Shaanxi/s2/2012
A/swine/Hebei/11/2018
A/swine/Hebei/47/2018
A/swine/Liaoning/BX68/2016
A/swine/Zhejiang/1/2007
A/swine/Shandong/275/2009
A/swine/Beijing/26/2008
A/swine/Fujian/58/2008
A/swine/Hubei/101/2009
A/swine/England/WVL7/1992
A/swine/Belgium/WVL2/1983
A/swine/Tianjin/01/2004
A/Dunedin/2/2000
A/Singapore/6/1986
A/swine/Shanghai/1/2005
A/swine/Guangdong/L6/2009
A/swine/Shandong/1112/2008
A/swine/Guangdong/322/2006
A/swine/Beijing/156/1991
A/swine/Wisconsin/1/1967
98
96
99
100
92
90
99
99
98
100
99
99
99
93
100
pdm/09 H1N1
Triple reassortant
Eurasian avian-like
Human seasonal
Classical swine
0.05

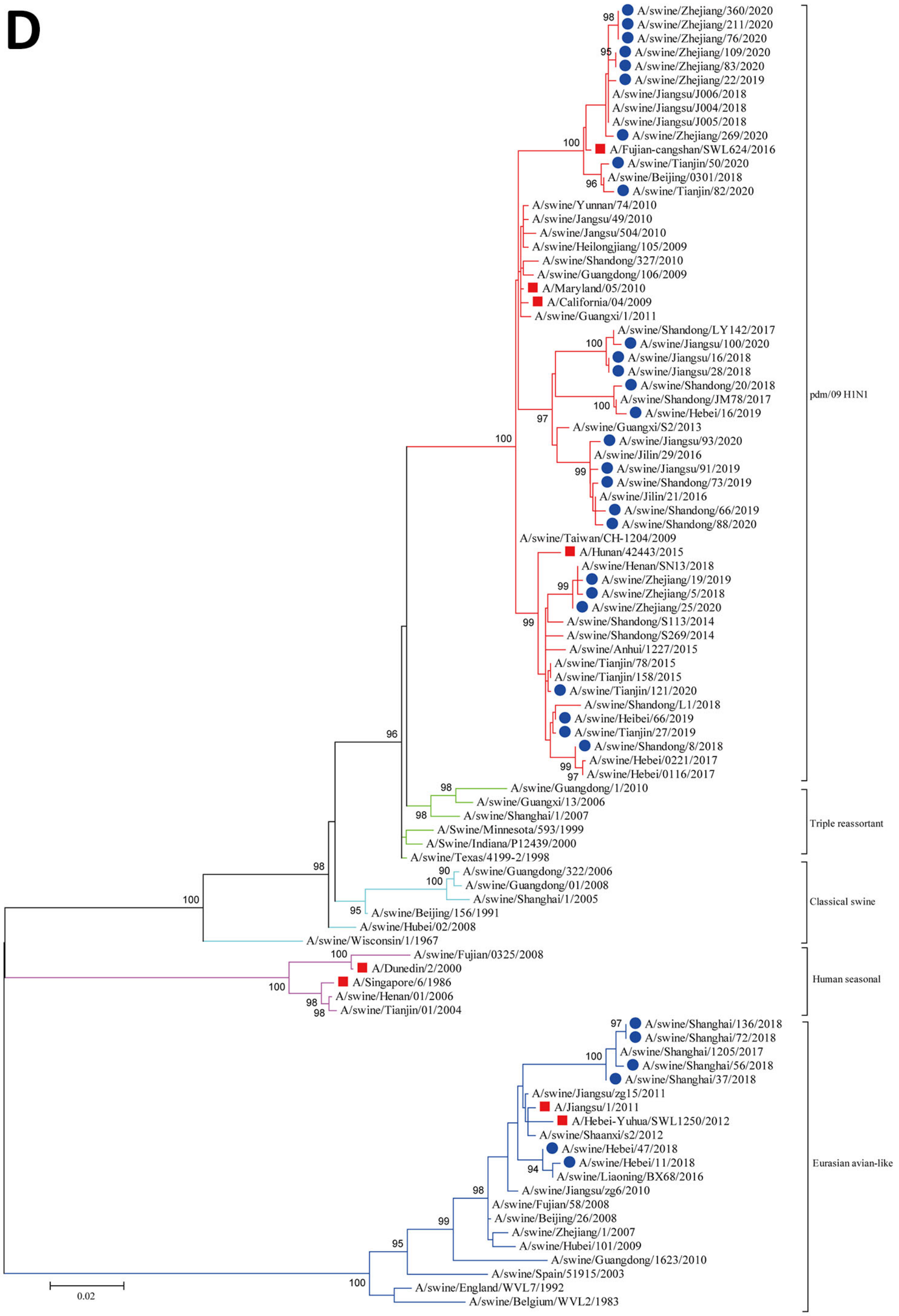
D
A/swine/Zhejiang/360/2020
A/swine/Zhejiang/211/2020
A/swine/Zhejiang/76/2020
A/swine/Zhejiang/109/2020
A/swine/Zhejiang/83/2020
A/swine/Zhejiang/22/2019
A/swine/Jiangsu/J006/2018
A/swine/Jiangsu/J004/2018
A/swine/Jiangsu/J005/2018
A/swine/Zhejiang/269/2020
A/Fujian-cangshan/SWL624/2016
A/swine/Tianjin/50/2020
A/swine/Beijing/0301/2018
A/swine/Tianjin/82/2020
A/swine/Yunnan/74/2010
A/swine/Jangsu/49/2010
A/swine/Jangsu/504/2010
A/swine/Heilongjiang/105/2009
A/swine/Shandong/327/2010
A/swine/Guangdong/106/2009
A/Maryland/05/2010
A/California/04/2009
A/swine/Guangxi/1/2011
A/swine/Shandong/LY142/2017
A/swine/Jiangsu/100/2020
A/swine/Jiangsu/16/2018
A/swine/Jiangsu/28/2018
A/swine/Shandong/20/2018
A/swine/Shandong/JM78/2017
A/swine/Hebei/16/2019
A/swine/Guangxi/S2/2013
A/swine/Jiangsu/93/2020
A/swine/Jilin/29/2016
A/swine/Jiangsu/91/2019
A/swine/Shandong/73/2019
A/swine/Jilin/21/2016
A/swine/Shandong/66/2019
A/swine/Shandong/88/2020
A/swine/Taiwan/CH-1204/2009
A/Hunan/42443/2015
A/swine/Henan/SN13/2018
A/swine/Zhejiang/19/2019
A/swine/Zhejiang/5/2018
A/swine/Zhejiang/25/2020
A/swine/Shandong/S113/2014
A/swine/Shandong/S269/2014
A/swine/Anhui/1227/2015
A/swine/Tianjin/78/2015
A/swine/Tianjin/158/2015
A/swine/Tianjin/121/2020
A/swine/Shandong/L1/2018
A/swine/Heibei/66/2019
A/swine/Tianjin/27/2019
A/swine/Shandong/8/2018
A/swine/Hebei/0221/2017
A/swine/Hebei/0116/2017
A/swine/Guangdong/1/2010
A/swine/Guangxi/13/2006
A/swine/Shanghai/1/2007
A/Swine/Minnesota/593/1999
A/Swine/Indiana/P12439/2000
A/swine/Texas/4199-2/1998
A/swine/Guangdong/322/2006
A/swine/Guangdong/01/2008
A/swine/Shanghai/1/2005
A/swine/Beijing/156/1991
A/swine/Hubei/02/2008
A/swine/Wisconsin/1/1967
A/swine/Fujian/0325/2008
A/Dunedin/2/2000
A/Singapore/6/1986
A/swine/Henan/01/2006
A/swine/Tianjin/01/2004
A/swine/Shanghai/136/2018
A/swine/Shanghai/72/2018
A/swine/Shanghai/1205/2017
A/swine/Shanghai/56/2018
A/swine/Shanghai/37/2018
A/swine/Jiangsu/zg15/2011
A/Jiangsu/1/2011
A/Hebei-Yuhua/SWL1250/2012
A/swine/Shaanxi/s2/2012
A/swine/Hebei/47/2018
A/swine/Hebei/11/2018
A/swine/Liaoning/BX68/2016
A/swine/Jiangsu/zg6/2010
A/swine/Fujian/58/2008
A/swine/Beijing/26/2008
A/swine/Zhejiang/1/2007
A/swine/Hubei/101/2009
A/swine/Guangdong/1623/2010
A/swine/Spain/51915/2003
A/swine/England/WVL7/1992
A/swine/Belgium/WVL2/1983
pdm/09 H1N1
Triple reassortant
Classical swine
Human seasonal
Eurasian avian-like
0.02

E

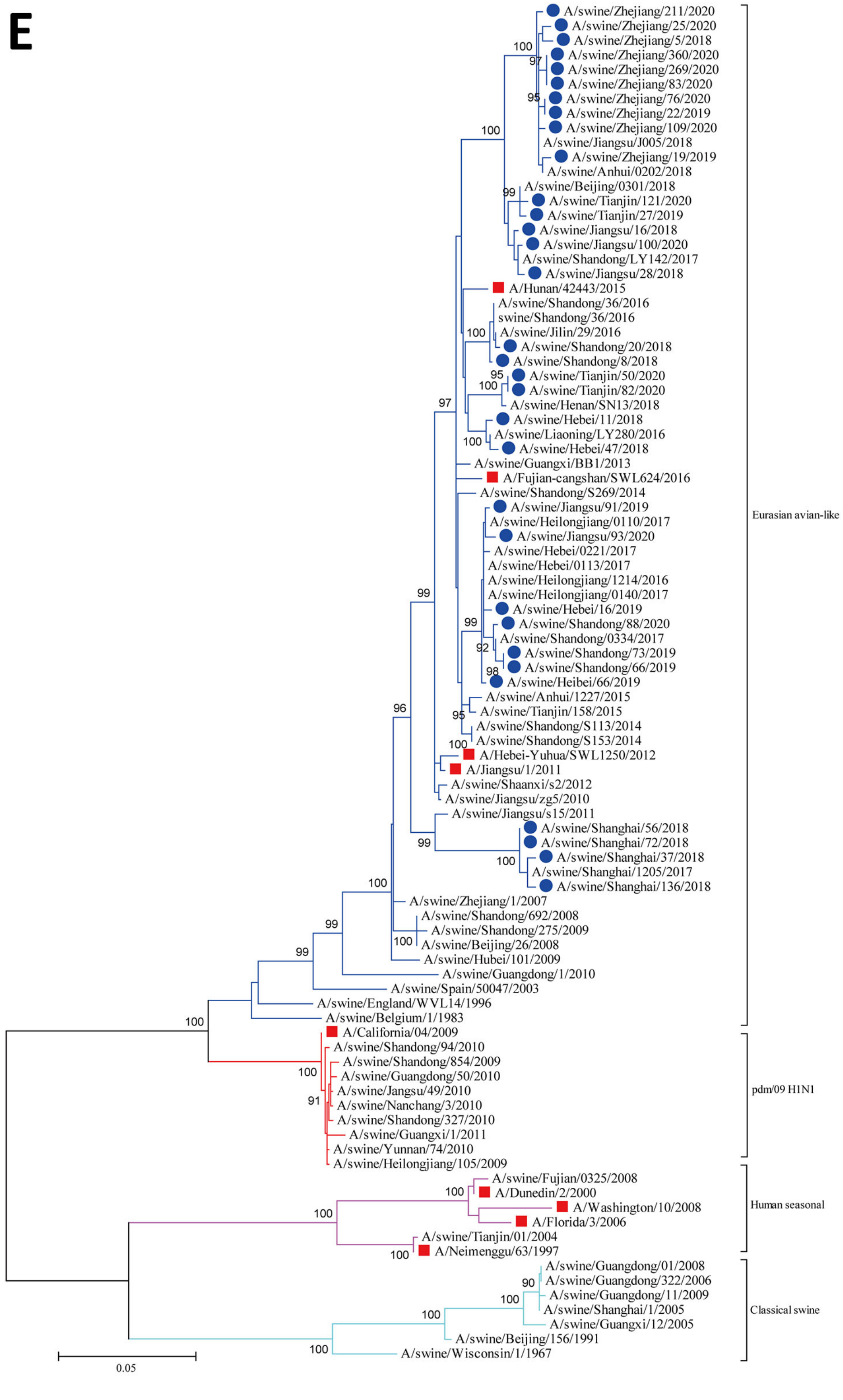
A/swine/Zhejiang/211/2020
A/swine/Zhejiang/25/2020
A/swine/Zhejiang/5/2018
A/swine/Zhejiang/360/2020
A/swine/Zhejiang/269/2020
A/swine/Zhejiang/83/2020
A/swine/Zhejiang/76/2020
A/swine/Zhejiang/22/2019
A/swine/Zhejiang/109/2020
A/swine/Jiangsu/J005/2018
A/swine/Zhejiang/19/2019
A/swine/Anhui/0202/2018
A/swine/Beijing/0301/2018
A/swine/Tianjin/121/2020
A/swine/Tianjin/27/2019
A/swine/Jiangsu/16/2018
A/swine/Jiangsu/100/2020
A/swine/Shandong/LY142/2017
A/swine/Jiangsu/28/2018
A/Hunan/42443/2015
A/swine/Shandong/36/2016
swine/Shandong/36/2016
A/swine/Jilin/29/2016
A/swine/Shandong/20/2018
A/swine/Shandong/8/2018
A/swine/Tianjin/50/2020
A/swine/Tianjin/82/2020
A/swine/Henan/SN13/2018
A/swine/Hebei/11/2018
A/swine/Liaoning/LY280/2016
A/swine/Hebei/47/2018
A/swine/Guangxi/BB1/2013
A/Fujian-cangshan/SWL624/2016
A/swine/Shandong/S269/2014
A/swine/Jiangsu/91/2019
A/swine/Heilongjiang/0110/2017
A/swine/Jiangsu/93/2020
A/swine/Hebei/0221/2017
A/swine/Hebei/0113/2017
A/swine/Heilongjiang/1214/2016
A/swine/Heilongjiang/0140/2017
A/swine/Hebei/16/2019
A/swine/Shandong/88/2020
A/swine/Shandong/0334/2017
A/swine/Shandong/73/2019
A/swine/Shandong/66/2019
A/swine/Heibei/66/2019
A/swine/Anhui/1227/2015
A/swine/Tianjin/158/2015
A/swine/Shandong/S113/2014
A/swine/Shandong/S153/2014
A/Hebei-Yuhua/SWL1250/2012
A/Jiangsu/1/2011
A/swine/Shaanxi/s2/2012
A/swine/Jiangsu/zg5/2010
A/swine/Jiangsu/s15/2011
A/swine/Shanghai/56/2018
A/swine/Shanghai/72/2018
A/swine/Shanghai/37/2018
A/swine/Shanghai/1205/2017
A/swine/Shanghai/136/2018
A/swine/Zhejiang/1/2007
A/swine/Shandong/692/2008
A/swine/Shandong/275/2009
A/swine/Beijing/26/2008
A/swine/Hubei/101/2009
A/swine/Guangdong/1/2010
A/swine/Spain/50047/2003
A/swine/England/WVL14/1996
A/swine/Belgium/1/1983
A/California/04/2009
A/swine/Shandong/94/2010
A/swine/Shandong/854/2009
A/swine/Guangdong/50/2010
A/swine/Jangsu/49/2010
A/swine/Nanchang/3/2010
A/swine/Shandong/327/2010
A/swine/Guangxi/1/2011
A/swine/Yunnan/74/2010
A/swine/Heilongjiang/105/2009
A/swine/Fujian/0325/2008
A/Dunedin/2/2000
A/Washington/10/2008
A/Florida/3/2006
A/swine/Tianjin/01/2004
A/Neimenggu/63/1997
A/swine/Guangdong/01/2008
A/swine/Guangdong/322/2006
A/swine/Guangdong/11/2009
A/swine/Shanghai/1/2005
A/swine/Guangxi/12/2005
A/swine/Beijing/156/1991
A/swine/Wisconsin/1/1967
Eurasian avian-like
pdm/09 H1N1
Human seasonal
Classical swine
0.05

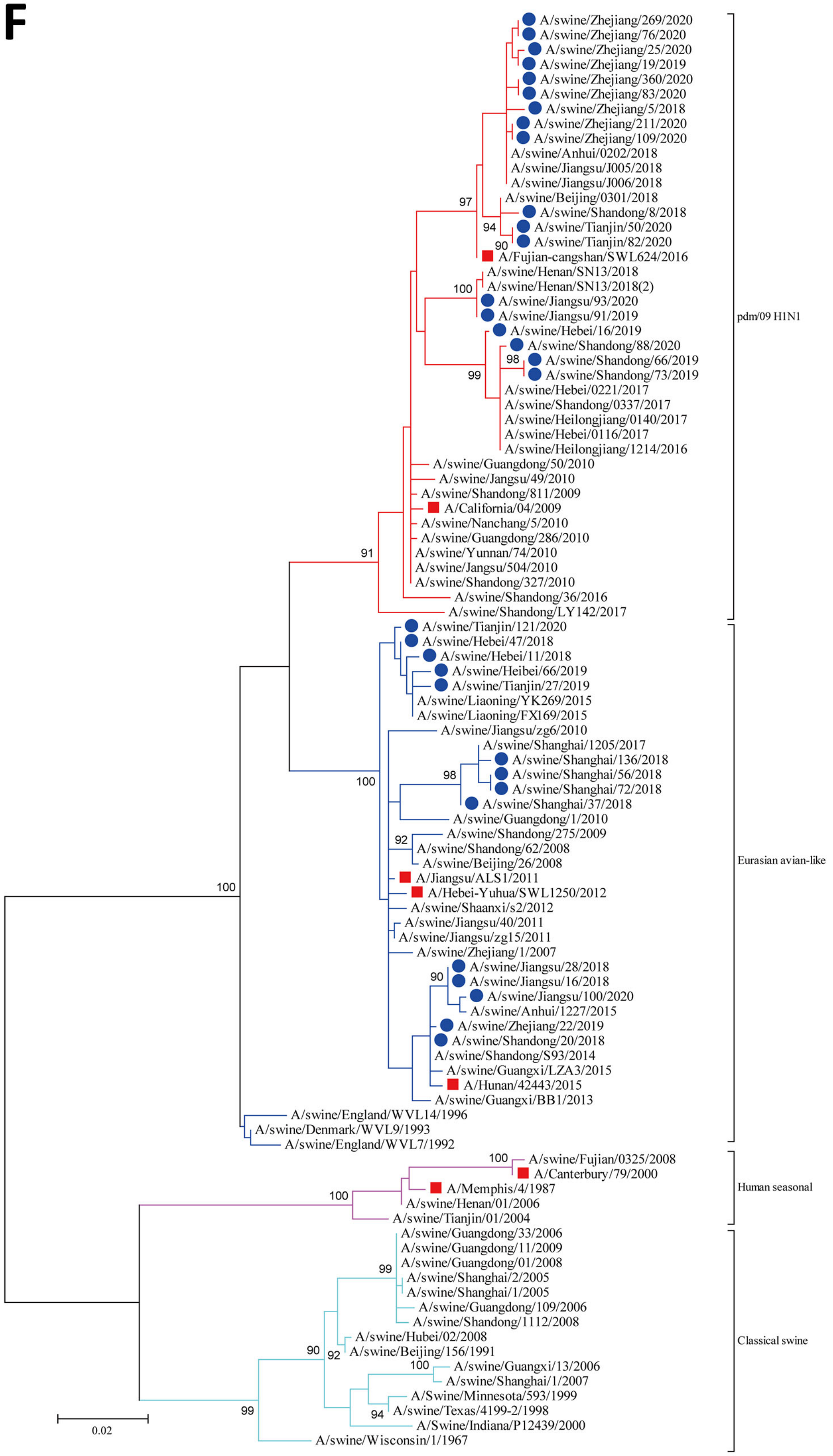
F
A/swine/Zhejiang/269/2020
A/swine/Zhejiang/76/2020
A/swine/Zhejiang/25/2020
A/swine/Zhejiang/19/2019
A/swine/Zhejiang/360/2020
A/swine/Zhejiang/83/2020
A/swine/Zhejiang/5/2018
A/swine/Zhejiang/211/2020
A/swine/Zhejiang/109/2020
A/swine/Anhui/0202/2018
A/swine/Jiangsu/J005/2018
A/swine/Jiangsu/J006/2018
A/swine/Beijing/0301/2018
A/swine/Shandong/8/2018
A/swine/Tianjin/50/2020
A/swine/Tianjin/82/2020
A/Fujian-cangshan/SWL624/2016
A/swine/Henan/SN13/2018
A/swine/Henan/SN13/2018(2)
A/swine/Jiangsu/93/2020
A/swine/Jiangsu/91/2019
A/swine/Hebei/16/2019
A/swine/Shandong/88/2020
A/swine/Shandong/66/2019
A/swine/Shandong/73/2019
A/swine/Hebei/0221/2017
A/swine/Shandong/0337/2017
A/swine/Heilongjiang/0140/2017
A/swine/Hebei/0116/2017
A/swine/Heilongjiang/1214/2016
A/swine/Guangdong/50/2010
A/swine/Jangsu/49/2010
A/swine/Shandong/811/2009
A/California/04/2009
A/swine/Nanchang/5/2010
A/swine/Guangdong/286/2010
A/swine/Yunnan/74/2010
A/swine/Jangsu/504/2010
A/swine/Shandong/327/2010
A/swine/Shandong/36/2016
A/swine/Shandong/LY142/2017
pdm/09 H1N1
A/swine/Tianjin/121/2020
A/swine/Hebei/47/2018
A/swine/Hebei/11/2018
A/swine/Heibei/66/2019
A/swine/Tianjin/27/2019
A/swine/Liaoning/YK269/2015
A/swine/Liaoning/FX169/2015
A/swine/Jiangsu/zg6/2010
A/swine/Shanghai/1205/2017
A/swine/Shanghai/136/2018
A/swine/Shanghai/56/2018
A/swine/Shanghai/72/2018
A/swine/Shanghai/37/2018
A/swine/Guangdong/1/2010
A/swine/Shandong/275/2009
A/swine/Shandong/62/2008
A/swine/Beijing/26/2008
A/Jiangsu/ALS1/2011
A/Hebei-Yuhua/SWL1250/2012
A/swine/Shaanxi/s2/2012
A/swine/Jiangsu/40/2011
A/swine/Jiangsu/zg15/2011
A/swine/Zhejiang/1/2007
A/swine/Jiangsu/28/2018
A/swine/Jiangsu/16/2018
A/swine/Jiangsu/100/2020
A/swine/Anhui/1227/2015
A/swine/Zhejiang/22/2019
A/swine/Shandong/20/2018
A/swine/Shandong/S93/2014
A/swine/Guangxi/LZA3/2015
A/Hunan/42443/2015
A/swine/Guangxi/BB1/2013
A/swine/England/WVL14/1996
A/swine/Denmark/WVL9/1993
A/swine/England/WVL7/1992
Eurasian avian-like
A/swine/Fujian/0325/2008
A/Canterbury/79/2000
A/Memphis/4/1987
A/swine/Henan/01/2006
A/swine/Tianjin/01/2004
Human seasonal
A/swine/Guangdong/33/2006
A/swine/Guangdong/11/2009
A/swine/Guangdong/01/2008
A/swine/Shanghai/2/2005
A/swine/Shanghai/1/2005
A/swine/Guangdong/109/2006
A/swine/Shandong/1112/2008
A/swine/Hubei/02/2008
A/swine/Beijing/156/1991
A/swine/Guangxi/13/2006
A/swine/Shanghai/1/2007
A/Swine/Minnesota/593/1999
A/swine/Texas/4199-2/1998
A/Swine/Indiana/P12439/2000
A/swine/Wisconsin/1/1967
Classical swine
97
94
90
100
98
99
91
100
98
92
90
100
100
100
99
90
92
100
94
99
0.02

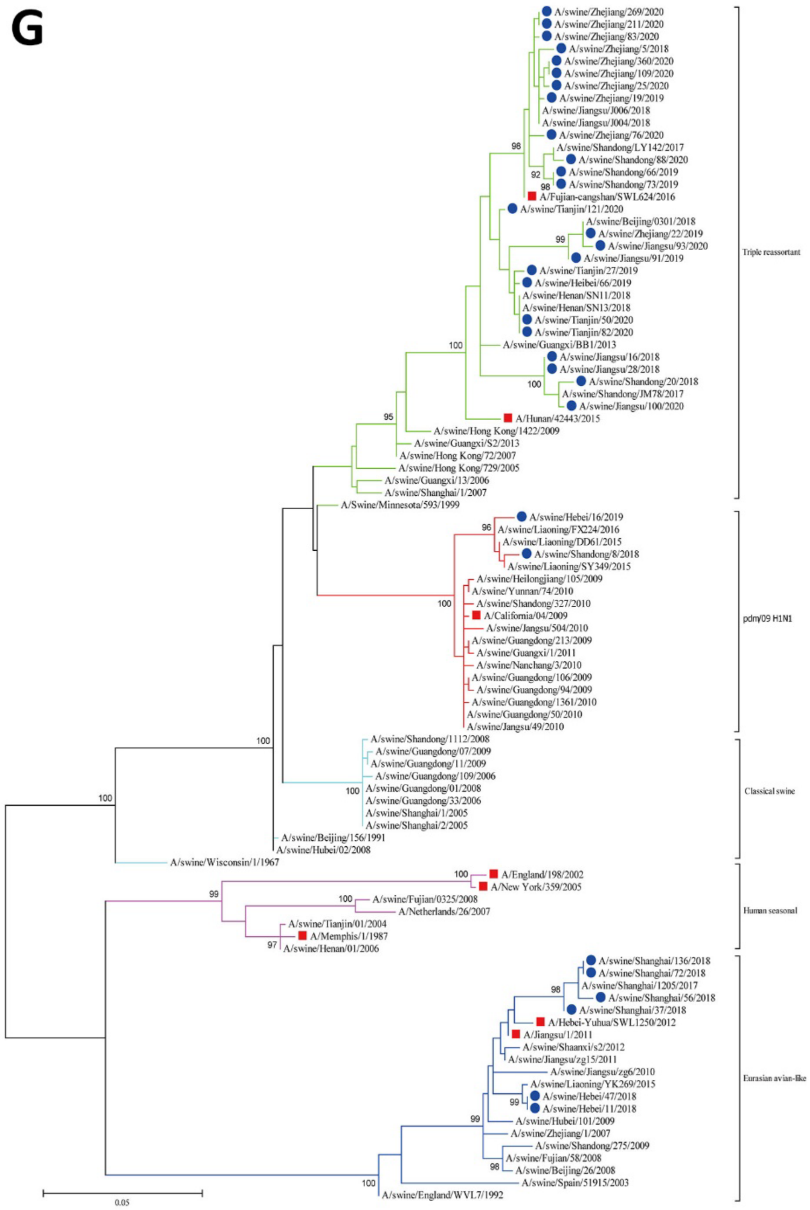


**Appendix Figure.** Maximum-likelihood phylogenetic tree of the A) PB2, B) PB1, C) PA, D) NP, E) NA, F) M, and G) NS genes. The phylogeny of available sequences of related viruses from GenBank and GISAID database (https://www.gisaid.org), and the sequences of 32 isolates in this study were inferred by using MEGA version 7 (https://www.megasoftware.net) under the general time-reversible plus gamma distribution model with 1,000 bootstrap replicates. Red triangles indicate reference sequences from humans. Blue circles indicate isolates sequenced in this study. Scale bar indicates substitutions per nucleotide.
